# Primary ovarian angiosarcoma: a rare and recognizable ovarian tumor

**DOI:** 10.1186/s13048-021-00771-7

**Published:** 2021-01-28

**Authors:** Hong Ye, Min Lin, Ruotong Li, Shuming Qin, Gang Hou, Hongzhi Chen, Xiaomei Li

**Affiliations:** Department of Pathology, Tai’an Central Hospital, No. 29, Longtan Road, Tai’an, 271000 China

**Keywords:** Angiosarcoma, Ovary, Pathology, Immunochemistry, Therapy

## Abstract

The diagnosis of primary angiosarcoma of ovary is still a challenge as it has no specific clinical symptoms and is easily confused with other malignant neoplasms in morphology. Here, we described a case of primary ovarian angiosarcoma and reviewed the literature. A 47-year-old female showed a left ovary mass. Grossly, the cut surface of the tumor was solid and gray-white with intermediate texture. Some areas were spongy and atropurpureus with a soft texture. Microscopically, the tumor cells were arranged into a variety of different structures with visible hemorrhage. Immunochemically, the tumor cells were positive for CD31, ERG, Fli1, D2–40 and vimentin in a strong and diffused manner. CD34 stain showed focal positivity. Epithelial markers (e.g. CK, CK7, CK8/18 and PAX8) were all negative. Negative immunostaining for SMA, S-100, P53 and calretinin also were detected. The proliferative index (Ki-67) was approximately 40%. After surgery, the patient was treated with radiotherapy, targeted therapy and immunotherapy. In the 9-month follow-up, the patient was survival without evidence of disease. The diagnosis of ovarian angiosarcoma required the careful observation of morphology and the reasonable application of immunohistochemistry. Targeted therapy and immunotherapy are the potential directions for the treatment of angiosarcoma.

## Introduction

Angiosarcoma, a rare soft tissue malignancy accounting for 1–2% of all soft tissue sarcomas, occurs in the skin tissues and soft tissues [1]. It is an infiltrative tumor with high rate of local recurrence and metastasis [2]. Such disease has been reported in liver, spleen, adrenal, heart, gastrointestinal tract and female genital tract (FGT) [3–8]. To our best knowledge, ovarian angiosarcoma is rare, responsible for about 1% of the ovarian malignancy [9–11]. Most of primary ovarian angiosarcomas are single onset, while partial cases present simultaneous teratoma or ovarian epithelial neoplasms [12].

The diagnosis of primary ovarian angiosarcoma is still a challenge as there are no specific clinical symptoms for these patients. Meanwhile, it is easily confused with other malignant neoplasms in morphology. In clinical settings, there is usually misdiagnosis of primary ovarian angiosarcomas due to high malignant degree, diverse clinical manifestations and rapid progress, which results in poor prognosis. In this study, we reported a case of primary ovarian angiosarcoma. Besides, a literature review was conducted to discuss its clinical features and pathological characteristics.

## Material and methods

The specimens were fixed in 10% buffered formalin after oophorosalpingectomy, followed by embedding in paraffin. The sections (4 μm) were stained using hematoxylin and eosin. The histological features were evaluated by two experienced pathologists. Immunohistochemistry stain was conducted with Ventana BenchMark XT automated IHC stainer (Roche, Basel, Switzerland). Sections treated with PBS served as negative control. The positive control was set using the specific tissues according to the manufacture’s instructions. Antibody information was given in Table 1. The patient signed the informed consent. The study protocols were approved by the Ethical Committee of Tai’an Central Hospital.

**Table 1:** Antibody information

Antibody	Clon	Source	Dilution
CK	AE1/AE3	Dako	1:100
CK7	E29	Dako	1:500
CK8/18	Cam5.2	Dako	1:200
Vimentin	V9	Dako	1:200
P53	DO-7	Dako	1:200
Calretinin	polyclone	Abcam	1:500
CD34	QBEnd/10	Dako	1:50
CD31	JC/70A	Dako	1:50
D2–40	D2–40	Abcam	1:200
ERG	ER111	Dako	1:50
Fli1	EPR4646	Abcam	1:200
PAX8	polyclone	Proteintech Group	1:800
S100	polyclone	Dako	1:2000
SMA	1A4	Dako	1:200
Ki67	MIB-1	Dako	1:50

## Results

### Clinical history

**Fig. 1:**
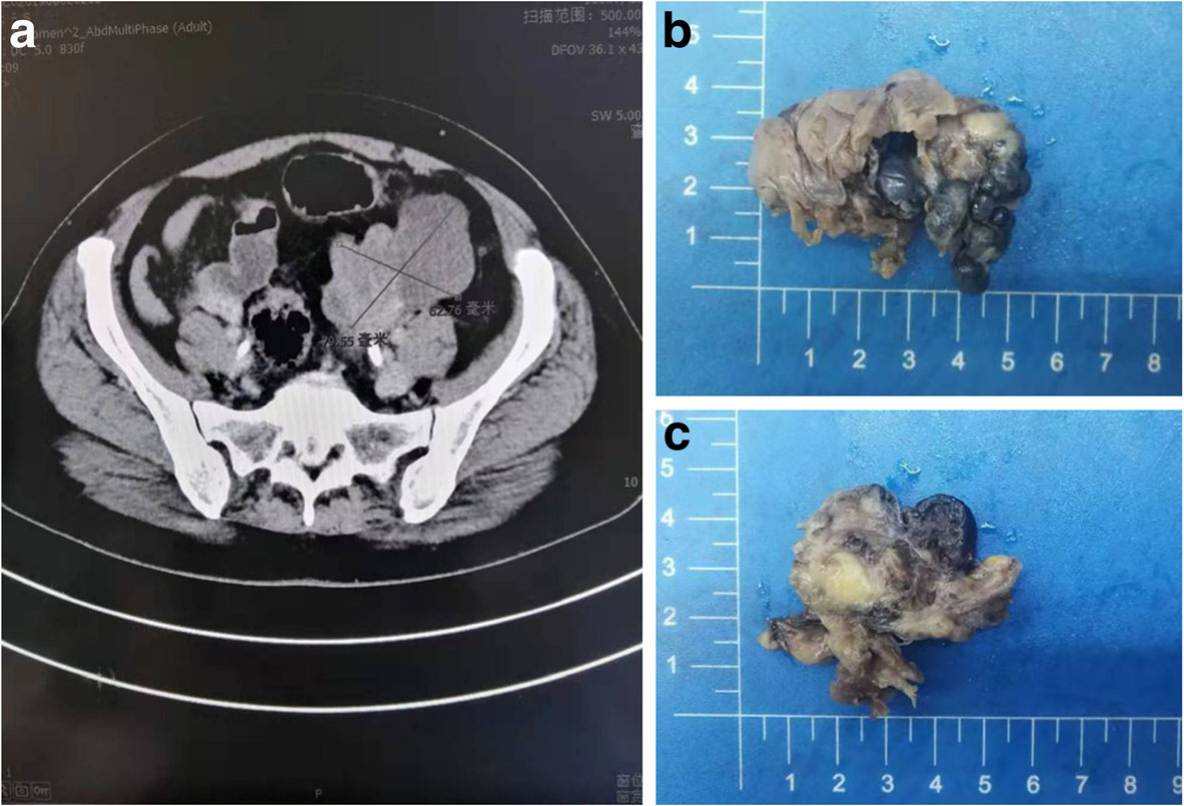
CT findings and macroscopic observation for the tumor mass. **a**; CT scan revealed an irregular soft tissue density mass shadow in the left appendix area, with a size of 7.9 × 6.2 cm. The mass was nodular and lobulated with clear edges. The unevenness of the lesion was significantly enhanced after enhanced scanning. **b**: Grossly, the left ovary was enlarged and sized 5.5 × 4.5 × 4 cm with nodular appearance and gray-white to atropurpureus surface. **c**: Some areas of the cut surface were solid and gray-white with intermediate texture, and some areas were spongy and atropurpureus with soft texture

A 47-year-old female (G2P2) presented to our department with a pelvic mass after physical examination about 10 days ago. The results of serological ovarian cancer markers were as follows: CA125, 9.456 U/ml (normal range: < 35 U/ml); CA19–9, 8.16 U/ml (normal range: < 39 U/ml); CA72–4, 2.666 U/ml (normal range: < 6.9 U/ml); CA15–3, 8.836 U/ml (normal range: < 25 U/ml); and CEA, 2.6 ng/ml (normal range: < 5.1 ng/ml). Color Doppler ultrasonography showed a mass (9 cm × 6.3 cm) with mixed echo and abundant blood flow signals of a low resistance index (RI) of 0.28 in left adnexa. CT scan revealed a mass shadow (7.9 cm × 6.2 cm) with irregular soft tissue density in the left appendix area. The mass was nodular and lobulated with clear edges. The uneven lesion was significantly enhanced after contrast enhanced scan (Fig. 1a). During the operation, the left ovary was occupied by a cystic-solid mass presenting multi-chamber and brown fluid. The mass was closely adhered to the surrounding peritoneum and rectum. No ascites was observed. There were no obvious abnormalities in the appearance of the right appendix, uterine and rectal lacunae, greater omentum and pelvic abdominal lymph nodes. The patient received total hysterectomy and bilateral salpingo-oophorectomy with pelvic and abdominal lymphadenectomy, omentectomy, and appendectomy. FIGO stage of the patient was IA. After surgery, the patient received 15 fractions of radiotherapy, 2 cycles of targeted chemotherapy using Olaparib (150 mg, b.i.d.), as well as immunotherapy. In the 9-month follow-up, the patient was survival with no evidence of recurrence.

### Pathological findings

Grossly, the left ovary showed enlargement (5.5 cm × 4.5 cm × 4 cm) with nodular appearance and a gray-white to atropurpureus surface (Fig. 1b). The cutting surface in some regions was solid in a color of gray-white with intermediate texture. In other regions, it was spongy and atropurpureus with soft texture (Fig. 1c).

**Fig. 2:**
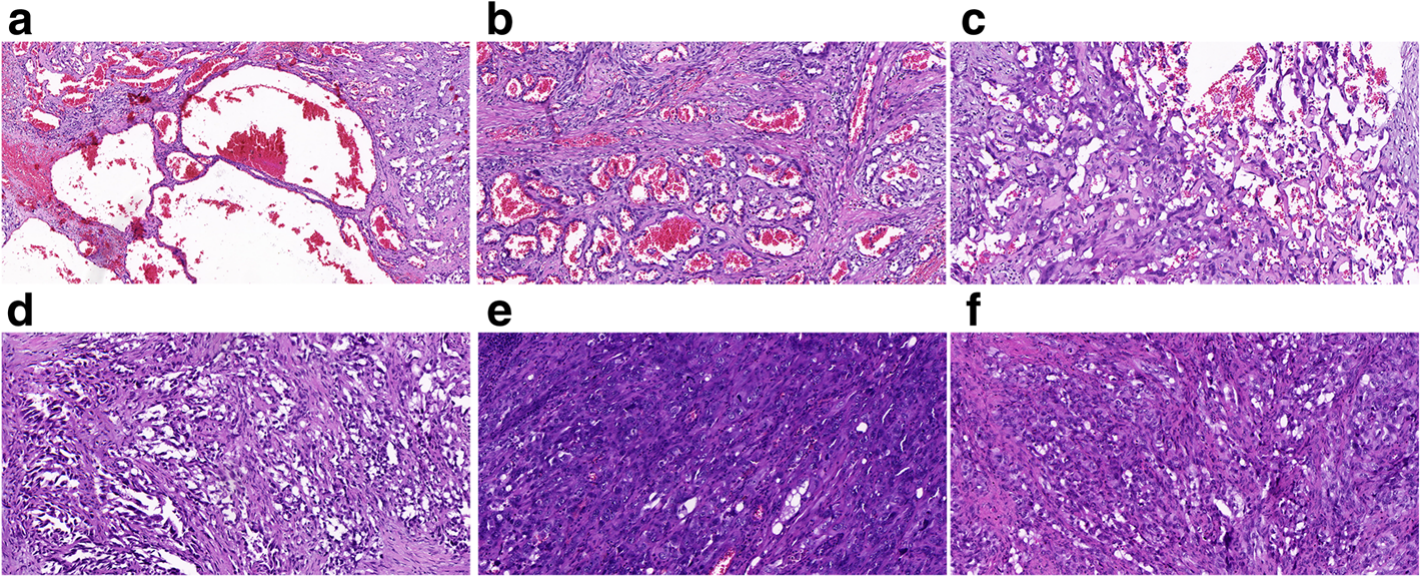
Pathological findings of the tumor. **a**: In well-differentiated areas, there were many vascular lumens of different sizes, dilated partly and filled with blood (4×); **b**: The lumens were lined with flattened or obese mildly atypical endothelial cells (10×); **c** and **d**: Some areas tumor cells were lined in an irregular labyrinth cavity structure, with the lumen expanding and anastomosing with each other. It was covered with one or more layers of swollen endothelial cells, showing papillary and boot-nail protruding into the lumen (10×). **e**: In the poorly differentiated areas, fusiform and epithelioid tumor cells formed solid nests without obvious channel and some tumor cells, which showed significant pleomorphism with vacuolated nuclei and obvious nucleoli scattered in patch. Mitotic were 5 to 10 per 10 high power field and included pathological karyokinesis (10×). **f**: Vacuoles cells that similar to adipoblast cells were observed in focal lesions (10×)

Microscopically, the tumor was located in the ovarian parenchyma and was poorly circumscribed with infiltrative growth pattern towards the stroma. The tumor cells were arranged into a variety of different structures with visible hemorrhage, including well-differentiated hemangiomatous areas, moderately differentiated fissure and communicating cystic tubular areas, and poorly differentiated solid patchy areas. In well-differentiated areas, there were many vascular lumens of various sizes with partial dilatation, which were filled with blood (Fig. 2a). The lumens were lined with flattened or obese mildly atypical endothelial cells (Fig. 2b). In some areas, tumor cells were lined in an irregular labyrinth cavity structure, with the expansion and anastomosis in the lumen. They were covered with one or more layers of swollen endothelial cells, with papillary and boot-nail protruding into the lumen (Fig. 2c and d). In the poorly differentiated areas, there were solid nests formed by fusiform and epithelioid tumor cells with no obvious channel. Partial tumor cells showed significant pleomorphism with vacuolated nuclei and obvious nucleoli scattered in patch. Mitotic cells (5–10 cells per 10 high power field) were seen including cells underwent atypical mitosis (Fig. 2e). Vacuoles cells that similar to adipoblast cells were observed in focal parts (Fig. 2f).

**Fig. 3:**
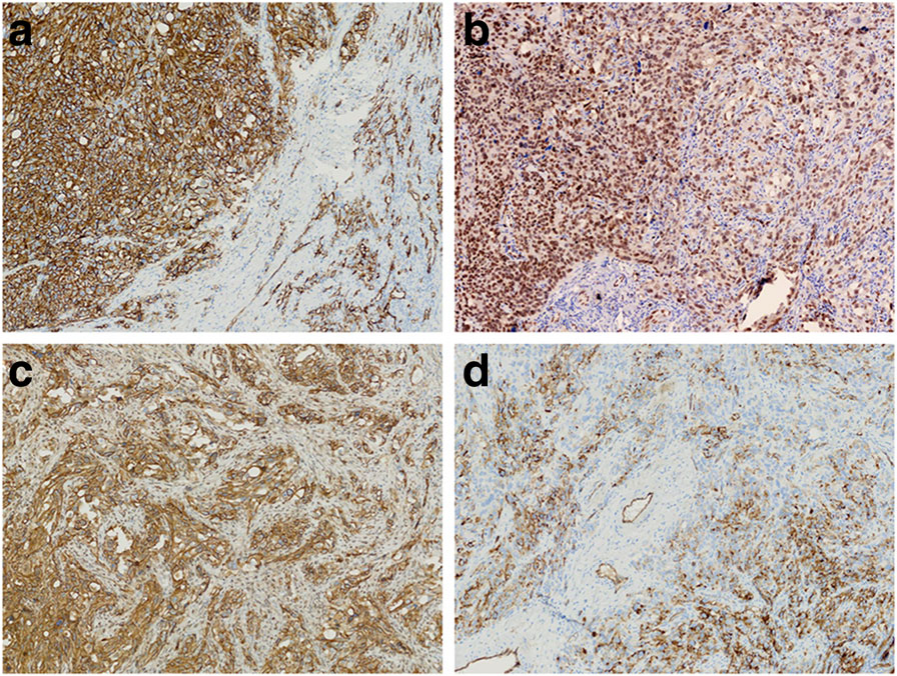
The tumor cells were positive for CD31 (**a**), ERG (B), D2–40 (**c**) in a strong and diffused manner. CD34 stain showed focal positivity (**d**) under a magnification of 20 ×

Immunochemically, the tumor cells were positive for CD31 (Fig. 3a), ERG (Fig. 3b), Fli1, D2–40 (Fig. 3c) and vimentin in a diffused manner. CD34 stain showed focal positivity (Fig. 3d). Besides, the epithelial markers including CK, CK7, CK8/18 and PAX8 were all negative. The immunostaining for SMA, S-100, P53 and calretinin also were negative. The proliferative index (Ki-67) was approximately 40%.

## Discussion

Angiosarcoma, commonly occurs in soft tissues, rarely presents in the FGT, especially in ovary. The incidence of primary ovarian angiosarcoma is 1/1,000,000 of ovarian malignant tumors and it may occur either as pure sarcoma or in combination with other ovarian tumors such as teratoma, mucinous cystadenocarcinoma, and dermoid cysts [12]. There were only 31 cases of primary ovarian angiosarcomas in the previous literatures [10, 12–34] . In this article, we reported one case with primary ovarian angiosarcomas, and then a comprehensive literature review was carried out to investigate the clinical features and prognosis of the tumor (Table 2). Primary angiosarcoma of ovary mainly occurred in premenopausal women with an averaged age of 33 years old. Only 3 cases occurred in postmenopausal women with the oldest one aged 81 years old [12, 25, 31]. In pediatric patients, the ovarian angiosarcoma was reported with the youngest age of 11 years old [27, 32–34]. Most patients had abdominal pain and distension, while the cases were accompanied by ascites or hemoperitoneum [25]. In most cases, the tumors were unilateral with the majority at the right ovary and 3 cases were bilateral [20, 27, 30]. The range of the size of the tumors was in a range of 2.1–30 cm.

**Table 2:** Summary of outcomes and findings of cases with primary pure angiosarcomas of the ovary reported

Literature	Case ID	Age, yr	Size, cm	Stage	Position	Follow up	Postoperative adjuvant therapy
Patel et al., 1991 [13]	1	42	Not available	IV	Right	DOD, 18 days	None
Cunningham et al., 1994 [14]	2	19	12 × 10	IV	Left	DOD, 7 mo	Doxo/ifos, 4 cycles; cisplat-num/etoposide, 1 cycle
Nara et al., 1996 [15]	3	33	4	IV	Right	DOD, 2 mo	None
Nielsen et al., 1997 [16]	4			I		NED	None
	5			I		5.5 yr–9 yr	
	6			I			
	7	20–32	6–13	I	Not available		
	8			III		DOD, 2 mo	
Furihata et al, 1998 [17]	9	46	21 × 16 × 13	Not available	Right	DOD, 9 mo	Cisplatin, 1 cycle; radiation
Lifschnitz-Mercer et al, 1998 [9]	10	25	13 × 11 × 5	III	Left	Recurrent disease, 18 mo+	Doxo/ifos, 3 cycles
Nucci et al, 1998 [18]	11	35		IV	Not available	DOD “quickly”	None
				I	Left		
	12	25	3.5–14	III	Not available	NED, 3 mo	
	13	42				DOD, 2 yr	
				I	Right		
	14	27				NED,14 mo	
Platt et al., 1999 [19]	15	40	11 × 8	IV	Left	NED, 2 mo	MAID, 4 cycles
Twu et al., 1999 [20]	16	40	Not available	IV	Bilateral	DOD, 7 mo	Doxo/ifos, 8 cycles
Davidson et al., 2005 [21]	17	19	18 × 15 × 15	III	Left	DOD, 1 yr	Doxo/ifos, 6 cycles
QuesenbErr et al., 2005 [22]	18	31	19 × 16 ×8.5	IC	Left	NED,1 yr	MAID, 3 cycles
Jha et al., 2005 [23]	19	28	20 × 25	I	Right	NED,10 mo	Doxo/ifos, 6 cycles
Vavilis et al., 2007 [24]	20	29	8 × 6	Not available	Right	Not available	None
Bradford et al., 2010 [25]	21	67	12 × 6 × 8	IIIC	Right	DOD,1 mo	Paclitaxel, 1 cycle
Serrano et al., 2010 [26]	22	23	14	IIIC	Left	NED,12 mo	Epirubicin/ifos, 6 cycles
Iljazovic et al., 2011 [27]	23	11	Left: 17 × 14 × 6 Right: 14 × 7 × 5	IIA	Bilateral	NED, 10 mo	Chemotherapy, 6 cycles
Bosmuller et al., 2011 [11]	24	81	30 × 18 ×12	I	Right	NED, 5 mo	Doxo, 4 cycles
Guseh et al., 2012 [28]	25	40	15 × 11 × 2	IIIC	Right	Recurrent disease, 18mo+	Doxo/ifos, 3 cycles
Yaqoob et al., 2014 [29]	26	41	7 × 6 × 2	IA	Left	Not available	None
Wu et al., 2014 [30]	27	45	Left: 7.1 × 4.7 Right: 2.1 × 1.4	IIIA	Bilateral	DOD, 30 mo	MAID, 6 cycles
Gaiolla et al., 2014 [31]	28	71	4.4	Not available	Right	DOD, 27mo	Gemcitabine/zoledronic acid, 2 cycles
Darre et al., 2017 [32]	29	12	17 × 14 × 9	II	Right	NED “not afford it”	None
Priyakumari et al., 2018 [33]	30	11	15 × 10 × 8	Not available	Right	NED “unwilling for treatment”	None
Pariury et al., 2019 [34]	31	11	Not available	Not available	Right	NED, 43 mo	Demcitabine/doxo, 12 cycles
Current case	32	47	7 × 4 × 4	I	Left	NED, 8 mo+	Olaparib, anti-PD-1

*NED* No evidence of disease; *DOD* Dead of disease; *yr* Year; *mo* Month. *MAID* Mesna + doxorubicin + ifosfamide + dacarbazine

Histopathological confirmation is essential for the final diagnosis of primary ovarian angiosarcoma. Generally, the histological characteristics of ovarian angiosarcoma were complex and diverse. Irregular vascular lumen structures could be seen in tumor tissues as the lumens confluent and communicated mutually. The cavity was covered with atypical cells [19]. Part of tumor cells was bosselated. In some cases, tumor cells showed solid flaky distribution, which showed cytologic atypia morphology with plump cytoplasm, eosinophilic feature, large nucleus, deep nuclear chromatin, as well as significant nucleoli and common mitotic figures. In addition, singe cells can perform as a vessel in some regions [35]. In our case, there was a mixture structure with well differentiated vascular area. The areas of the vascular cavity communicated with each other and lined with spike-like heterotypic cells, as well as distribution of the solid flake spindle cells and epithelioid cells area. The variety of tissue structure and cell morphology reflected the complicacy and multiformity of morphology of angiosarcoma, which caused difficulties in clinical diagnosis.

CD31 is expressed in more than 90% of angiosarcomas, and the positive rate of CD34 in angiosarcomas is 50–60% [36]. In poorly differentiated angiosarcomas, the sensitivity and specificity of CD31 were more effective than that of CD34. In recent years, the new antibody (e.g. ERG and Fli1) mainly expressed in the vascular endothelium can be used to mark benign and malignant tumors of vascular origin with high specificity. Nevertheless, it could also be expressed in some tumors of non-vascular origin [37, 38]. Therefore, the combination of CD31, CD34, ERG, Fli1 and other antibodies was more conducive to accurate diagnosis. In this case, the tumor cells were positive for CD31 and CD34 stain with focal positivity. D2–40 can be expressed in some patients with angiosarcomas [39]. Our results showed that D2–40 was diffuse and strongly positive, which further validated the vascular origin of the tumor.

Highly differentiated angiosarcoma should be differentiated from benign hemangioma, especially anastomosing hemangiomas. Grossly, ovarian hemangioma usually located in the ovarian medulla with polycystic, spongy and bleeding areas. The clear boundary between tumor and surrounding tissue was an important indicator for the distinguishing the hemangioma and highly differentiated angiosarcoma. The vascular cavities of anastomosing hemangiomas showed anastomosis with each other and lined with flattened or hobnail-like endothelial cells. There was no atypia among these cells, while highly differentiated angiosarcoma cells showed cytological atypia and invasion of surrounding tissues [40]. In addition, juvenile cellular hemangioma was an important differential diagnosis. In a previous study, Prus reported a case of infantile hemangioma in the ovary of a neonate [41]. The tumor was composed of blood vessels of different sizes lined with swollen endothelial cells, together with eosinophilic cytoplasm and vacuolated nuclei. Small nucleoli and karyokinesis were seen. No pathological karyokinesis were observed.

Poorly differentiated angiosarcomas are often composed of spindle cells and epithelioid cells with significant atypia, which is easily confused with sarcomatoid cancer and soft tissue sarcomas. Sarcomatoid carcinomas usually present well-differentiated carcinomatous components expressing epithelial immune markers (e.g. ck, ck7, and ck8/18). Meanwhile, vascular markers (e.g. CD31) were negative, which could distinguish them from angiosarcomas. For the other soft tissue sarcomas such as leiomyosarcoma, the tumor cells were usually arranged in beam of the spindle cells, with flake of epithelioid cells focally. The present of structure of vascular compartment suggested the diagnosis of angiosarcoma. A panel of immunohistochemical antibodies including SMA, Desmin, CD31 and ERG were conducive to the identification of these tumors.

Tumor cells of high-grade ovarian serous carcinoma and clear cell carcinoma are often arranged like adenoids or glandular cysts, which protruded into the glandular cavity with eosinophilic cytoplasm. There was obviously allotypic and hyperchromatic nuclei, as well as more mitosis. Moreover, tumor cells in some regions were arranged in a solid pattern. However, well-differentiated hemangio-like areas were accompanied by significant bleeding, irregular intersecting tubular-cystic structures. Immunohistochemically, these cells were negative for CK and CK7 and positive for CD31 and CD34, which contributed to the confirmation of the diagnosis. The yolk sac tumor presented multicystic, adenoid and cranny structures, and there were nail-like cells in the capsular space and adenoid cavity that were easily confused with the angiosarcoma. However, in the yolk sac tumor, there were Schiller-Duval body and the porous edema. The immunohistochemisty results for SALL4, AFP and Glypican-3 were positive (Table 3). The angiosarcoma may originate from the teratoma. Therefore, extensive sampling was required to investigate the benign teratoma components.

**Table 3:** Immunohistochemisty of angiosarcoma and differential diagnosis

Immunohistochemisty	Angiosarcoma	Serous carcinoma	Clear cell carcinoma	Yolk sac tumor
CK	+/−	+	+	+/−
CK7	–	+	+	–
SALL4	–	–	–	+
CD34,CD31, ERG	+	–	–	–
WT-1	–	+	–	–
P53	–	+	–	–
NapsinA	–	–	+	–
HNF-1β	–	–	+	–
AFP	–	–	–	+
Glypican-3	–	–	–	+
D2–40	+	–	–	–

It is necessary to exclude metastatic hemangiosarcoma before the diagnosis of primary ovarian sarcoma of the ovary. In our case, the patient underwent a comprehensive physical examination. No tumor was found in other parts of the body and there was no history of angiosarcoma. Therefore, the neoplasm in ovary was considered as primary angiosarcoma.

Cytogenetically, the expression of *FLT1* and *AKT3* in the angiosarcomas patients was up-regulated. Recent studies demonstrated that the *PTPRB* and *PLCG1* genes involved in angiogenesis were mutated in angiosarcomas. In addition, 9% of cases showed aberrant *CIC* and 7% of the cases showed *KDR* mutation. *MYC* gene amplification was confirmed to play key roles in secondary angiosarcomas. Further studies are required to investigate the genetic mutations of most primary angiosarcomas [42].

To date, the major treatment options for angiosarcoma include surgical debulking and post-operative adjuvant chemotherapy and radiotherapy. In a previous study, surgical resection was performed in the majority of cases, while some patients underwent adjuvant chemotherapy after surgery. Common chemotherapy regimens for primary ovarian angiosarcoma include the MAID regimen, as well as ifosfamide and doxorubicin, as well as gemcitabine and cisplatin [28]. Jha et al. reported a 28-year-old woman received adjuvant chemotherapy with ifosfamide + doxorubicin for fertility preservation, which finally delivered a healthy living baby [23]. Currently, clinical staging is considered as the most important factor affecting the prognosis of patients. In the previous study, stages were obtained for 27 patients in the literature [9, 11, 13–16, 18–23, 25–32], including 11 patients with stage I, 2 patients with stage II and 14 patients with stage III and IV. Finally, follow-up information of 10 cases (stage I: 8 cases; stage II: 2 cases) was obtained. All the patients were followed up for 3 months to 9 years, and were confirmed with disease-free survival. For the 14 cases at stage III, 9 cases were died about 18 days or 30 months after diagnosis (Table 2).

In this case, the patient underwent 15 fractions of radiation and adjuvant targeted therapy with the PARP inhibitor (i.e. Olaparib). In addition, PD-L1 determination was performed in the tumor samples, which indicated PD-L1 positivity. Anti-PD-1 immunotherapy was also given to her. No evidence of disease recurrence was noticed in the 9-month follow-up. In recent years, PARP inhibitors have been approved and applied in the treatment of epithelial ovarian cancer, with satisfactory efficiency [43]. In a multi-centered phase I study, the combination of trabectedin and olaparib showed promising efficiency for treating soft tissue sarcoma [44]. In future, further studies are required to investigate the roles and efficiency of PARP inhibitors in treating ovarian angiosarcoma. The effects of anti-PD-1 in the treatment of angiosarcoma are still lacking of large experimental studies. Sindhu [45] et al. reported a case of nasal angiosarcoma showing satisfactory efficiency after anti-PD-1 therapy. This indicated that the anti-PD-1 immunotherapy may serve as a promising treatment option for treating angiosarcoma.

However, after taking the efficiency of such agent in treating other tumors into considering, its application in treating angiosarcoma is still promising [42].

## Conclusion

Ovarian angiosarcoma is very rare with no specific clinical symptoms. The prognosis of patients with advanced stage is still poor. The diagnosis of poorly differentiated angiosarcoma is highly relied on the identification of communicating and typical vascular-like structures. Immunopositivity for a specific endothelial marker (e.g. CD31, CD34, EGR, or Fli1) is a diagnostic prerequisite. Complete surgical resection and postoperative adjuvant chemoradiotherapy are routine treatment methods. In future, targeted therapy may be a new type of exploratory therapy.

## Data Availability

All the data were available upon appropriate request
